# In vivo Monitoring of Serotonin by Nanomaterial Functionalized Acupuncture Needle

**DOI:** 10.1038/srep28018

**Published:** 2016-06-15

**Authors:** Yu-Tao Li, Li-Na Tang, Yong Ning, Qing Shu, Feng-Xia Liang, Hua Wang, Guo-Jun Zhang

**Affiliations:** 1 School of Laboratory Medicine, Hubei University of Chinese, 1 Huangjia Lake West Road, Wuhan 430065, China; 2 Hubei Provincial Collaborative Innovation Center of Preventive Treatment by Acupuncture and Moxibustion, 1 Huangjia Lake West Road, Wuhan 430065, China

## Abstract

Acupuncture treatment is amazing but controversial. Up to now, the mechanism of treating diseases by acupuncture and moxibustion is still unclear, especially the occurrence of the molecular events in local acupoints. Herein, we report an extremely stable microsensor by modifying carbon nanotube (CNT) to the tip surface of acupuncture needle and applying this CNT-modified acupuncture needle for real time monitoring of serotonin (5-HT) *in vivo*. To stabilize CNT modification on the needle tip surface, poly(3,4-ethylenedioxythiophene)(PEDOT) was employed as glue water to stick CNT on the needle. The detection limit of the CNT-modified needle was found to be approximately 50 nM and 78 nM in the PBS and the cell medium, respectively. In addition, the needle showed good selectivity to some inflammatory mediators and some electroactive molecules. For the first time, the CNT-modified needle could be directly probed into rat body for real time monitoring of 5-HT *in vivo*, showing a great potential for better understanding the mechanism of acupuncture treatment.

Acupuncture treatment is an old but amazing technology in Traditional Chinese Medicine, which is gaining popularity as a non-pharmacological option in pain medicine^1^,^2^,^3^. The present studies have demonstrated that the cellular signal is produced and delivered through the pathway of channels (meridians) during the needling manipulation process, leading to downstream effects that activate certain cellular pathways and facilitate healing^4^,^5^. It is well known that analgesic effects of acupuncture are systemic. Currently, many attentions have been paid on studying the connections of these signal molecules with the needle stimulation at the central and peripheral level^6^,^7^,^8^. However, the local molecular event is taken place while acupuncture is locally manipulated in body acupoints. As a result, it is of great significance to understand the local responses to acupoint stimulation.

Mast cells are known as immune cells that play an active role in the immune response through degranulation to release biological substances including tryptase, histamine, and serotonin (5-HT) *et al*. The mast cells have been reported to play an important role in the local response in acupoints. It has been found through both human and animal research studies that acupoints contain a significantly higher concentration of mast cells than sham point^9^,^10^,^11^,^12^. While the acupuncture needle is manually inserted into the acupoints, the degranulation of the mast cells increases remarkably, resulting in change of the bioactive substances. Wu, *et al*. revealed the response of cutaneous nerve fibers and mast cells to manual acupuncture stimulation in acupoint Hegu (LI4) by employing immunohistochemistry analysis method, in which the cells were conducted *in vitro*^13^. However, Not only does the fluorescence-based approach require expensive instruments, but also the fluorescent dyes are harmful and undergo photobleaching. Therefore, it is desirable to develop a method capable of monitoring the chemical substances concentration changes released by mast cells in local acupoints *in vivo* and in real time.

Serotonin (5-HT), as a representative substance released by mast cells, plays a pivotal role in alleviating pain. As the degranulation of the mast cells increases, the local 5-HT concentration rises significantly in the process of the needling manipulation process. So far, 5-HT levels in the central nervous and in the blood of platelets have been assayed by extraction, followed by high precision liquid chromatography (HPLC) analysis^14^ and enzyme-linked immunosorbent assay (ELISA)^15^. However, the 5-HT levels in acupoint tissues are in a state of dynamic change, making these techniques less feasible at this site. Moreover, these methods are slow, require extensive sample treatment, and do not provide real-time and *in vivo* quantitative information. Electrochemical methods include advantages of low cost, high sensitivity and short measurement time. Most importantly, they can monitor the dynamic change of signal molecules *in vivo* and in real time^16^,^17^,^18^,^19^,^20^. In our last work, we have successfully fabricated graphene-modified acupuncture needle by using layer-to-layer electrochemical deposition of Au nanoparticles and graphene on the tip surface of acupuncture needle, and used it for sensitive detection of neurotransmitters via electrochemistry^21^. As an electroactive compound, 5-HT can also be studied by electrochemical methods.

Carbon nanotubes (CNTs), with the properties of high modulus of elasticity, high aspect ratios, and excellent thermal and electrical conductivities, offer a wide variety of potential applications for various technological fields. Especially in the case of electrochemical conduction, CNT electrodes show extraordinary electrical conductivity, chemical stability, resistance against surface fouling, and good biocompatibility^22^,^23^,^24^,^25^. However, CNTs are hard to be integrated into the tip of acupuncture needle surface and are easy to peel off when the CNT-modified needle is inserted into body tissues. Poly(3,4-Ethylenedioxythiophene)(PEDOT) has been considered as the most promising conducting polymer because its ordered and well-defined chemical structure offers outstanding conductivity and stability. It has been reported that the electrical properties of biosensor electrodes can be significantly improved by surface coating with PEDOT^26^,^27^. In addition, previous reports have shown that CNTs can be incorporated into conducting polymers, such as polypyrrole^28^ and PEDOT^29^ to form composite materials with enhanced properties, including stability. Therefore, it is expected that the CNT doped with PEDOT (PEDOT/CNT) as a coating material may preserve the biocompatibility of PEDOT and CNTs, and exhibit enhanced long-term stability.

Herein, we report a unique nanosensing platform by modifying CNTs on the tip surface of acupuncture needle to prepare CNT-modified acupuncture needle (CNT/AN), and using it for real time monitoring 5-HT *in vivo* via electrochemistry. As illustrated in Fig. 1, CNT and PEDOT are assembled on the tip surface of the acupuncture needle, and 5-HT can be monitored in local acupionts *in vivo* by using the modified acupuncture needle. In this work, we take the PEDOT as the glue water to stick CNTs on the tip surface of acupuncture needle. Afterwards, PEDOT/CNT films are electrochemically coated on the tip of acupuncture needle, and their morphology and electrochemical properties are characterized. Results show that PEDOT and CNT have a synergistic effect towards the electrocatalytic reaction of 5-HT. The PEDOT/CNT/AN presented in this work exhibits an efficient electrochemical response with high resistance against chemical fouling. The standard working curves and the real time working curves are investigated. Finally we use the as-prepared PEDOT/CNT/AN for real time and *in vivo* monitoring of 5-HT level in acupoint *zusanli* (ST 36).

## Results and Discussion

### Properties of the PEDOT/CNT-modified acupuncture needle

**Figure 2:**
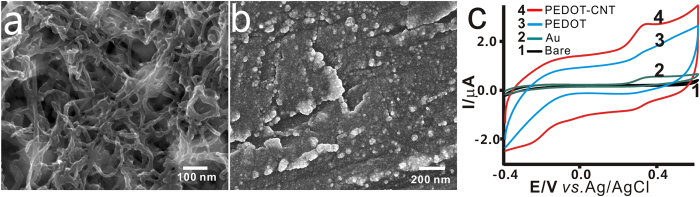
The basic properties of the PEDOT/CNT-modified acupuncture needle. (**a**) SEM pictures of PEDOT packaged with CNTs; (**b**) SEM pictures of pure PEDOT; (**c**) The CV graphs of different materials modified acupuncture needle: (1) bare acupuncture needle, (2) Au layer, (3) PEDOT, (4) PEDOT/CNT.

Real time and *in vivo* monitoring of the concentration changes of signal molecules in acupoints requires a sensitive, stable and low noise electrode in order to ensure the working efficiency and to minimize the disturbance of the electrical signals^30^. As we all know, preparation of CNT electrodes by drops is commonly used. However, CNTs on the electrode surface are unstable and easy to peel off, especially when the CNT-modified electrode is inserted into the tissue. To avoid this, we made PEDOT as the glue water to stick CNTs on the tip of acupuncture needle for preparing a stable sensor. Before modification, the bare needle was sprayed with a thin Au layer for the purpose of an ideal modification at the next step. Then, the dispersible CNTs mixture with EDOT was electrochemically deposited on the tip of needle by electropolymerization. Figure 2a,b displays scanning electron microscopy (SEM) images of acupuncture needle after PEDOT/CNT and pure PEDOT modification. As seen in Fig. 2a, CNTs were tightly wrapped on the acupuncture needle tip by PEDOT. The CNTs deposited onto the acupuncture needle could give rise to a large surface area and good electrochemical properties compared with the pure PEDOT. The electrochemical performances of the needles, modified with different nanomaterials, were investigated by CV in the presence of 10 μM 5-HT. As presented in Fig. 2c, the pristine needle and PEDOT-modified needle had no distinct redox peak (Fig. 2c(1,3)), while irreversible electron redox behavior was observed on the CNT-modified acupuncture needle. The greatly enhanced peak current and oxidative peak separation were strong indicatives of the catalytic properties of Au or the PEDOT/CNT films towards the redox reaction of 5-HT (Fig. 2c(2, 4)). It is clear that CNT or the Au layer alone can catalyze the electrochemical reaction of 5-HT. But the PEDOT doped with CNTs can significantly enhance the catalytic property.

The PEDOT/CNT-modified needle showed larger current response than the Au and PEDOT-modified acupuncture needle, which is ascribed to the fact that CNTs with high effective surface area could be beneficial not only for enhancing electrochemical currents of diffusing electroactive species, but also for allowing the loading of high-density electrochemically active biomolecules. Thus the electrochemical response was significantly improved^31^,^32^. Moreover, PEDOT, as a high conduction polymer, could greatly increase the conductivity of the fabricated needle. The electrodeposited PEDOT/CNT film combines the excellent catalytic properties of PEDOT and CNTs, showing a synergistic effect towards the electrocatalytic reaction of 5-HT.

### Stability of the PEDOT/CNT modified acupuncture needle

**Figure 3:**
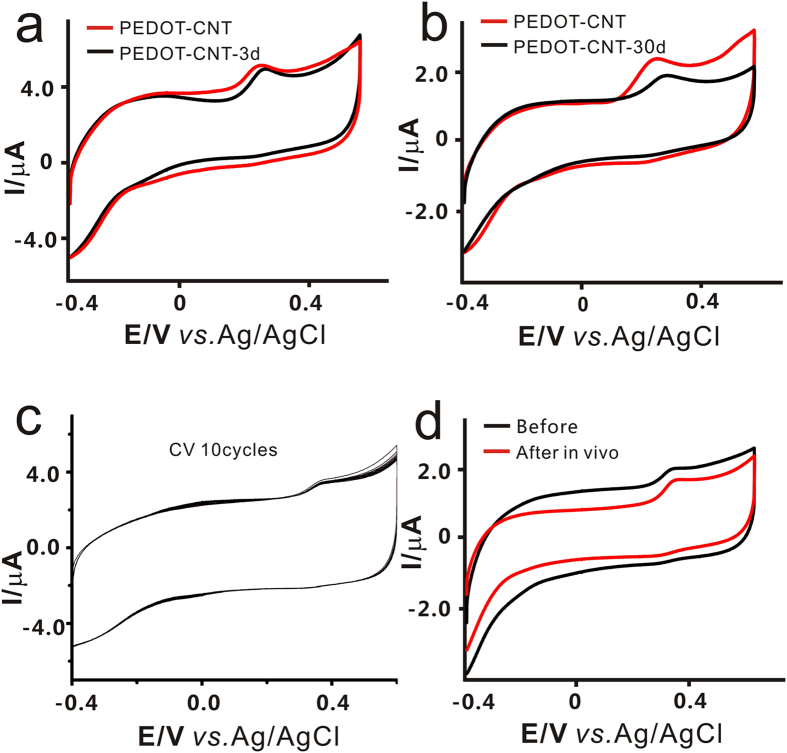
Stability of the PEDOT/CNT-modified acupuncture needle. (**a,b**) The CV graphs of PEDOT/CNT/AN before and after placed in air for 3 days(**a**) and 30 days(**b**), respectively ; (**c**) The CV graphs for acupuncture needle with 10 cycles; (**d**) The CV graphs for modified acupuncture needle before and after tested *in vivo*.

To investigate the stability of the prepared PEDOT/CNT/AN, the method of electrochemical CVs was again employed. First, the as-prepared sensor needles were placed in air at room temperature for 3 and 30 days, respectively, and their properties of CVs in 10 μM 5-HT solution were then tested. From Fig. 3a,b, we could see that the electrochemical performance of the as-prepared needles was hardly affected, when they were placed for only 3 days. Even after they were placed for 30 days, the electrochemical performance only decreased 20.34% ± 1.45(SD). Then, the modified needles were immersed into 50 μM 5-HT aqueous solution and scanned for 10 cycles. It was seen that almost each cycle overlapped with others on the modified needles (Fig. 3c). In order to further demonstrate the stability of PEDOT/CNT/AN for real time and *in vivo* measurement, the modified needle was inserted into ST 36 tissue and the measurement was conducted *in vivo*. The electrochemical performance of the needle was found to only decrease 22.13% ± 4.65(SD) (Fig. 3d). The results demonstrate that the as-prepared sensing needle is stable enough to be applied to work in complex environment and *in vivo*. All above-mentioned experiments indicate that the prepared sensing needle is extremely stable and overcomes the disadvantages of using an adsorption strategy to coat a polymer as the sensing membrane, which is easy to peel off.

### Determination of 5-HT

**Figure 4:**
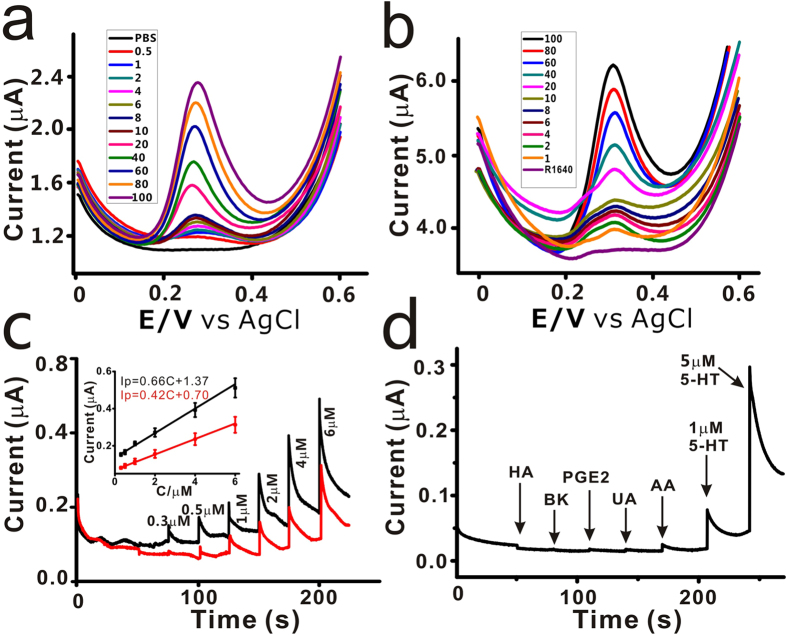
Quantitative analysis of 5-HT by PEDOT/CNT/AN. (**a,b**) The DPVs versus increasing 5-HT concentrations of 0, 1, 2, 4, 6, 8, 10, 20, 40, 60, 80 and 100 μM in the PBS and cell medium RPMI 1640; (**c**) Amperometric curves of PEDOT/CNT/AN to a series of 5-HT concentrations at a potential of +0.4 V. Inset: The calibration curve to different concentrations in cell medium (red line) and PBS solution (black line); (**d**) Selective profiles of the PEDOT/CNT/AN to interferences in the PBS, in which all interference species were at the concentration of 50 μM. Operating potential, 0.4 V vs Ag/AgCl.

The quantitative analysis of 5-HT was investigated via both DPV and amperometric I-T methods by applying various concentrations of 5-HT on the PEDOT/CNT/AN at pH 7.4. To further adjust the detection environment close to the human body, a cell medium (R1640) with serum was taken as the complex sample for amperometric detection. The amperometric detection of 5-HT with the PEDOT/CNT/AN was performed at a constant potential of 400 mV. The DPV responses of different concentrations of 5-HT on the PEDOT/CNT/AN in PBS solution and the cell medium (R1640) were presented in Fig. 4a,b, in which the DPV current signals increased along with the increased 5-HT concentrations. Representative amperometric I-T curves showing responses of the PEDOT/CNT/AN upon successive addition of various concentrations of 5-HT in PBS and R1640 were illustrated in Supplementary Fig. 1a,b (each concentration was repeated 3 times), from which we could see that the sensor needle exhibited a clear response to 5-HT at 0.1 μM and 0.2 μM, respectively.

The sensitivity of the PEDOT/CNT/AN in the R1640 (0.42 μA/μM, Fig. 4c) was about 64% of that in PBS solution (0.66 μA/μM, Fig. 4c), which may result from the partial adsorption of undesired compounds in the cell medium onto the active surface tip of the acupuncture needle. The linear relationship ranging from 0.3 to 6 μM between the peak currents and 5-HT concentrations was represented by Ip(μA) = 0.42 C (μM) + 0.70 (in R1640) and Ip(μA) = 0.66 C (μM) + 1.37 (in PBS solution). The detection limit was calculated to be about 50 nM and 78 nM in the PBS and the cell medium (R1640), respectively, which was estimated using 3σ/S (σ is standard deviation of the blank signal and S is the slope of the fit line shown in the inset of Fig. 4c). As seen in Table 1, compared with other electrochemical sensors for the determination of serotonin, the CNT-modified acupuncture needle obtained a low detection limit, which is comparable with that reported previously^33^,^34^,^35^,^36^,^37^,^38^,^39^.

The selectivity of PEDOT/CNT/AN towards 5-HT in PBS was further studied by investigating various interfering species. In this experiment, some other inflammatory mediators that are closely related to the pain, including histamine (HA), bradykinin (BK) and prostaglandin E_2_ (PGE2) were chosen. Some other electroactive molecules, such as ascorbic acid (AA) and uric acid (UA), were also investigated. The response of the commonly coexisting interferences was recorded at the PEDOT/CNT/AN sensor. As shown in Fig. 4d, successive addition of histamine, BK and PGE2 with the concentration of 50 μM exhibited negligible significant response. Similarly, the same concentration of electroactive AA and UA showed negligible significant response. However, the sensor needle indeed showed a clear response upon addition of 1 μM 5-HT. Further addition of 5 μM 5-HT led to an evident response, indicating that the sensor needle is highly selective and can be used for 5-HT detection in acupoints, and capable of avoiding the interference.

### *In vivo* monitoring of serotonin in acupuncture point

**Figure 5:**
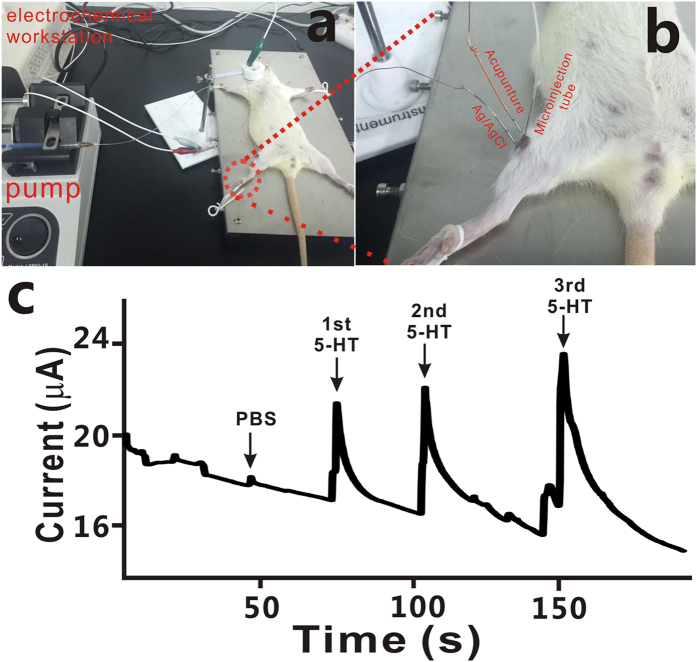
*In vivo* monitoring of 5-HT. (**a**) The photograph of real time and *in vivo* experiment after the rat was intraperitoneal anesthesia; (**b**) A zoomed picture; (**c**) Real time recording of the inserted PEDOT/CNT/AN in ST 36 upon repeated injection of 10 μL,10^−5^ M of 5-HT. Operating potential, 0.5 V vs Ag/AgCl.

As previously reported^9^,^10^,^11^,^12^, the degranulation of the mast cell increases while the needle is manipulated, making the serotonin levels greatly increased in local acupoints. Currently, acupuncture therapy is being widely debated in the medical community as a potential complementary treatment for many diseases. One of challenges to achieving a consensus over the use of acupuncture in a medical environment is to reveal the underlying molecular mechanisms of acupuncture, so real-time and *in vivo* monitoring of 5-HT concentration in acupuncture point is of great importance. In this work, a proof-of-concept experiment was performed, aiming at establishing a method of monitoring 5-HT level in real time and *in vivo*. The experimental procedure was carried out as follows. First, approximately 400 g of Wistar rat was put under general intraperitoneal anesthesia with 0.1 g/mL chloral hydrate and placed in an animal operating table (Fig. 5a,b). Electrochemical measurements were performed with a computer-controlled electrochemical analyzer and connected with the as-prepared PEDOT/CNT-modified acupuncture needle for both *in vitro* and *in vivo* measurements. These added solutions (PBS or 5-HT) were delivered from gas-impermeable syringes and pumped through Teflon plastic pipe by a microinjection pump. The PEDOT/CNT/AN was polarized at +0.5 V for *in vivo* amperometric monitoring of 5-HT.

The final goal of this work is to test the actual concentration of serotonin in ST 36. To investigate whether the functionalized acupuncture needle has the ability, we tried to directly monitor serotonin in ST 36 by pumping high K^+^ (80 mM) as the stimulant. But unfortunately, the signals of serotonin were not distinguishable from the control, as shown in Supplement Fig. S2. It is probably that the oxidation peak is not very obvious due to interference of the extremely complex environment *in vivo*, and uncertainty of serotonin concentration in acupoint.

Instead, to verify whether the functionalized acupuncture needle has the capability of real time monitoring of 5-HT *in vivo*, 5-HT was pumped into the acupoint ST 36 and measured *in vivo* as a proof-of-concept experiment. Prior to *in vivo* monitoring of 5-HT in the ST 36 acupoint of Wistar rats, PBS solution was first injected into acupoint by a microinjection pump for providing a better ionic solution, and the PBS solution did not provoke any change in the baseline current except for some disturbance(Fig. 5c). As depicted in Fig. 5c, with local microinfusion of 10 μL of 5-HT (10 μM) into the acupoint through a micropump, the current response was clearly visible. Through 3 times microinjection of 10 μL of 5-HT (10 μM) in the same place of ST 36 acupoint, we could see the evident amplitude response. Due to signal accumulation, the current amplitude increased temporally, indicating that the as-prepared PEDOT/CNT/AN is quite stable and able to detect the neurotransmitters *in vivo*.

In addition, it is reported that the primo vascular system, proposed as the anatomical structure of acupoints and meridians, also has a high density of mast cells^40^,^41^. Experiments were conducted to test whether the measurement is sensitive enough in the anatomical structure corresponding to acupoints, to the primo nodes in peritoneal cavity which has high concentration of mast cells as reported. The organ surface-primo vessels (PVs) in the abdominal cavity were obtained according to the literature under a stereomicroscope. As indicated in the yellow arrow in Fig. S3, the primo nodes were found to connect with PVs (Fig. S3a–c). But these primo nodes were quite small and could not be fixed *in vivo*. In addition, the sensing needle was too large to be inserted into the primo nodes for *in vivo* detection. So we collected these primo nodes and placed them in PBS solution (Fig. S3d). Then, we wrapped the primo nodes around the tip surface of the sensing needle with an attempt to collect electrochemical signals of 5-HT released from mast cells after high K^+^ stimulation (Fig. S3e). As indicated in Supplementary Fig. 4, the current response was clearly visible after the primo nodes tissue was stimulated by the high K^+^ solution. While the high K^+^ solution was applied to the same sensor needle without the primo nodes, hardly any increase in current was seen. Since the primo nodes contained rich mast cells, we could observe the oxidation peak of 5-HT signals by employing such a way. As reported, 5-HT released from enterochromaffin cells (ECs) is greatly calcium dependent. Most of different cell types can be depolarized by elevating the external potassium concentration^42^,^43^. When high K^+^ solution was pumped on the primo nodes tissue, high K^+^ triggered the cell membrane depolarization, inducing an action potential. Such an action potential opened the Ca^2+^ channels, making Ca^2+^ in-flowing and causing a remarkable release of 5-HT. Although we can’t directly insert the acupuncture needles into the primo nodes for *in vivo* detection, such an experiment indicates a direct measurement of serotonin on tissue, further demonstrating the sensor’s capability.

This work reported for the first time that the functional acupuncture needle could be directly probed into rat body for real time monitoring of the signal molecules in acupoints. Moreover, it was capable of withstanding fouling during 5-HT detection *in vivo*. Although the amount of serotonin produced by acupuncture therapy may not be detected out in the real sense, it is still a great progress compared with the previous work. There are indeed some difficulties existing. The accurate concentration of serotonin in acupoints has not been reported yet. In addition, the environment in living body is extremely complex, making real-time and *in vivo* detection more difficult.

## Conclusion

In summary, we have developed a novel acupuncture needle-based biosensor by modifying CNTs on the tip surface of acupuncture needle for real time and *in vivo* monitoring of serotonin. This work offered a new and reliable method for preparation of acupuncture needle-based sensor, providing a significant technical support and broad vision in the study of acupuncture, and promoting the development of traditional Chinese acupuncture and moxibustion medicine. Not only did the prepared acupuncture needle show good sensitivity and stability, but also it could be used for direct detection of signal molecules in complex cell medium solution, even *in vivo*. Compared with the previous work, a great progress has been made in this work, and in the next step we will move forward to differentiation of signal molecules in local acupoints and in sham points in the human body.

## Methods

### Materials and apparatus

Acupuncture needles were purchased from Suzhou medical supplies factory Co. Ltd. (Suzhou, China). 5-HT, DA and AA were obtained from Aladdin (Shanghai, china). 3, 4-Ethylenedioxythiophene (PEDOT) was purchased from Sigma-Aldrich. Multi-walled CNTs with the diameter of 10–20 nm and length of 10–30 μm were purchased (purity > 95 wt%) from Xianfeng nanomaterials company (Nanjing, China). All reagents used were analytical grade, and Milli-Q (Waters) water was used in all processes. The electrochemical experiment was carried out with the CHI660D electrochemical workstation (CH Instruments) and performed in a three-electrode system. Scanning electron microscopy images were obtained from a Zeiss Ultra Plus FE-SEM (Zeiss, Germany). All measurements were carried out at a room temperature.

### Electrochemical deposition of PEDOT/CNT

Electrochemical polymerization of PEDOT/CNT was performed as literature report^44^. Briefly, 10 mg CNTs were added into 5 mL of 0.02 M PEDOT aqueous solution. The solution was ultrasonicated for 2 h before polymerization. The potential for the polymerization was set at 1.2 V (vs. the Ag/AgCl reference electrode), and the polymerization time was 30 s unless otherwise stated. After polymerization, the modified electrode was washed thoroughly with water and dried at room temperature, and the prepared electrode was denoted as PEDOT/CNT.

### Electrochemical detection of 5-HT

To carry out the electrochemical detection, CV, DPV and I-T methods were employed in a three-electrode cell in 1× PBS solution. CVs were performed in the range of −0.4 ~ 0.6 V at a scan rate of 100 mV/s. The electrochemical parameters for DPVs were: 0~ 0.6 V, step potential, 0.004 V; modulation amplitude, 40 mV; pulse width, 0.05 s; pulse period, 0.2 s.

### *In vivo* measurements in acupoint

Wistar rats were purchased from the Experimental Animal Center of Centers for Disease Control and Prevention of Hubei Provincial, of which an average weight of 300 to 400 g was used. Animals were put under general anesthesia with 0.1 g/ml chloral hydrate and placed in an animal operating table. Limbs were fixed up with a rope. ST 36 was located 5 mm below and lateral to the anterior tubercle of the tibia; at this point, the needles were inserted perpendicularly at 3–5 mm^45^,^46^. In order to prevent CNT layer from falling off, cannula was first inserted into ST 36. Then the as-prepared needle was inserted into the casing. The reference electrode was placed nearby the working electrode in the same way. The syringe needle was used to exogenously microinfuse the aqueous solutions of PBS or 5-HT into the ST 36 of rats. These solutions were delivered from gas-impermeable syringes and pumped through Teflon plastic tubing by a microinjection pump (LSP02-1B, Baoding Lange, China). After that, two experimental protocols were performed: (i) the direct injection of PBS into the acupuncture point for providing the electrolyte and (ii) the direct injection of 10 μM 5-HT into the acupuncture point, in order to mimic an endogenous release of 5-HT. All experimental protocols conducted on these samples were approved by the Animal Experimental Committee of Hubei University of Chinese Medicine. All experiments on animals were carried out in accordance with the approved guidelines.

## Additional Information

**How to cite this article**: Li, Y.-T. *et al*. In vivo Monitoring of Serotonin by Nanomaterial Functionalized Acupuncture Needle. *Sci. Rep.* **6**, 28018; doi: 10.1038/srep28018 (2016).

## Supplementary Material

Supplementary Information

## Figures and Tables

**Figure 1:**
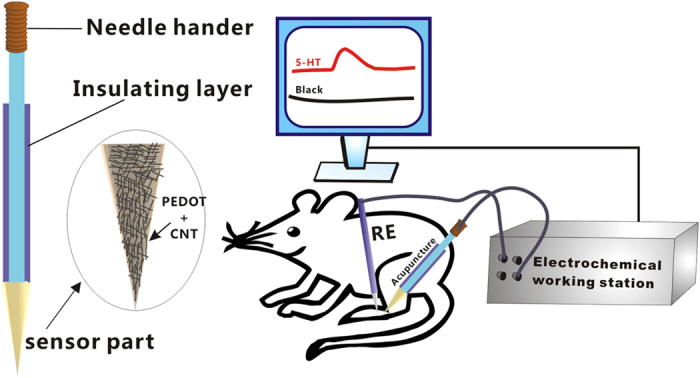
Schematic diagram of real time and *in vivo* monitoring of 5-HT by means of the PEDOT/CNT-modified acupuncture needle.

**Table 1:** Comparison of different electrochemical sensors for the determination of serotonin.

Electrode	Detection limit (μM)	Reference
FTO-PEDOT-PSS^a^	0.23	33
GC-PEDOT-SWCNT^b^	0.03	34
GC-MWCNTs-CHT^c^	0.08	35
GC-Ni(OH)_2_-CNT^d^	0.003	36
GC-G-g-PLA-Pd^e^	0.08	37
GC-ERGO-P^f^	0.03	38
GC-rGO/PANI-AuNPs^g^	0.0117	39
AN-PEDOT-CNT^h^	0.05	This work

^a^Fluorine doped tin oxide-poly (ethylenedioxythiophene)-poly(sodium 4-styrenesulfonate).

^b^Glassy carbon-polyethylenedioxythiophene single-walled carbon nanotube.

^c^Glassy carbon-carbon nanotube-chitosan.

^d^Glassy carbon-Ni(OH)_2_-carbon nanotube.

^e^Glassy carbon-graphene oxide grafted poly(lactic acid) with palladium nanopar-ticles.

^f^Glassy carbon-electrochemically reduced graphite oxide-porphyrin.

^g^Glassy carbon-reduced graphene oxide-polyaniline gold nanoparticles.

^h^Acupuncture needle- polyethylenedioxythiophene carbon nanotube.
