# Supplementary material for: In vivo Monitoring of Serotonin by Nanomaterial Functionalized Acupuncture Needle
Source: Sci Rep. 2016 Jun 15;6:28018. doi: 10.1038/srep28018 (PMC4908407; doi:10.1038/srep28018)
Supplement: Supplementary Information [file srep28018-s1.pdf]

Supporting Information

In vivo Monitoring of Serotonin by Nanomaterial Functionalized Acupuncture Needle

Yu-Tao Li[1], Li-Na Tang[1], Yong Ning[1], Qing Shu [2], Feng-Xia Liang[2], Hua Wang[2], Guo-Jun Zhang[1, 2*]

[1]School of Laboratory Medicine, Hubei University of Chinese,

1 Huangjia Lake West Road, Wuhan 430065, China

[2]Hubei Provincial Collaborative Innovation Center of Preventive Treatment by Acupuncture and Moxibustion, 1 Huangjia Lake West Road, Wuhan 430065, China

*Corresponding author: Tel: +86-27-68890259, Fax: +86-27-68890259

Email: zhanggj@hbtcm.edu.cn

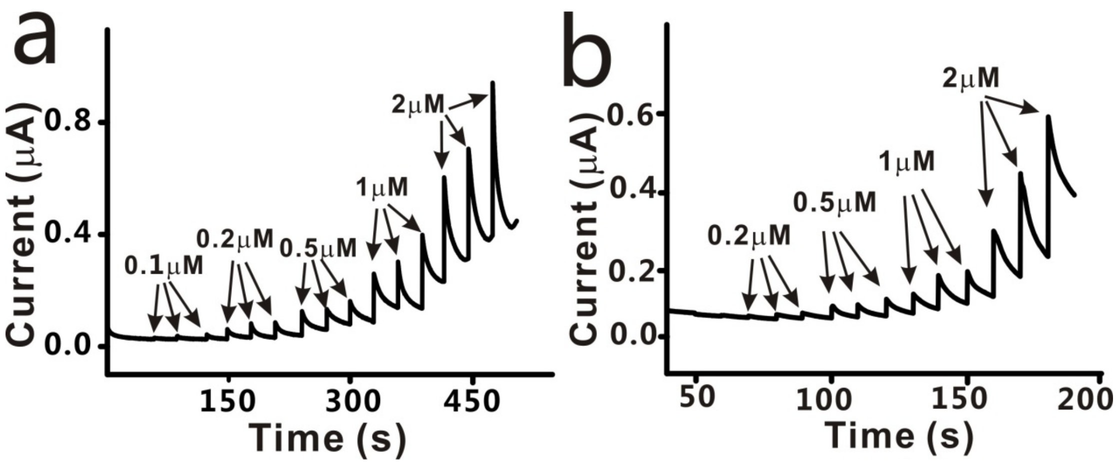

Fig. S1. (a-b) Amperometric curves of PEDOT/CNT/AN to a series of 5-HT concentrations in a stirred PBS solution and cell medium.

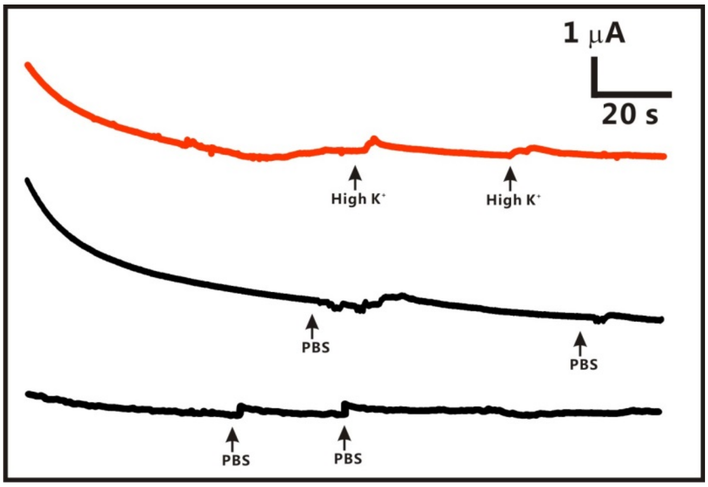

Fig. S2. Real-time monitoring of serotonin in ST36. The red line was stimulated by high K^+, and the black lines were stimulated by 1×PBS solution as control.

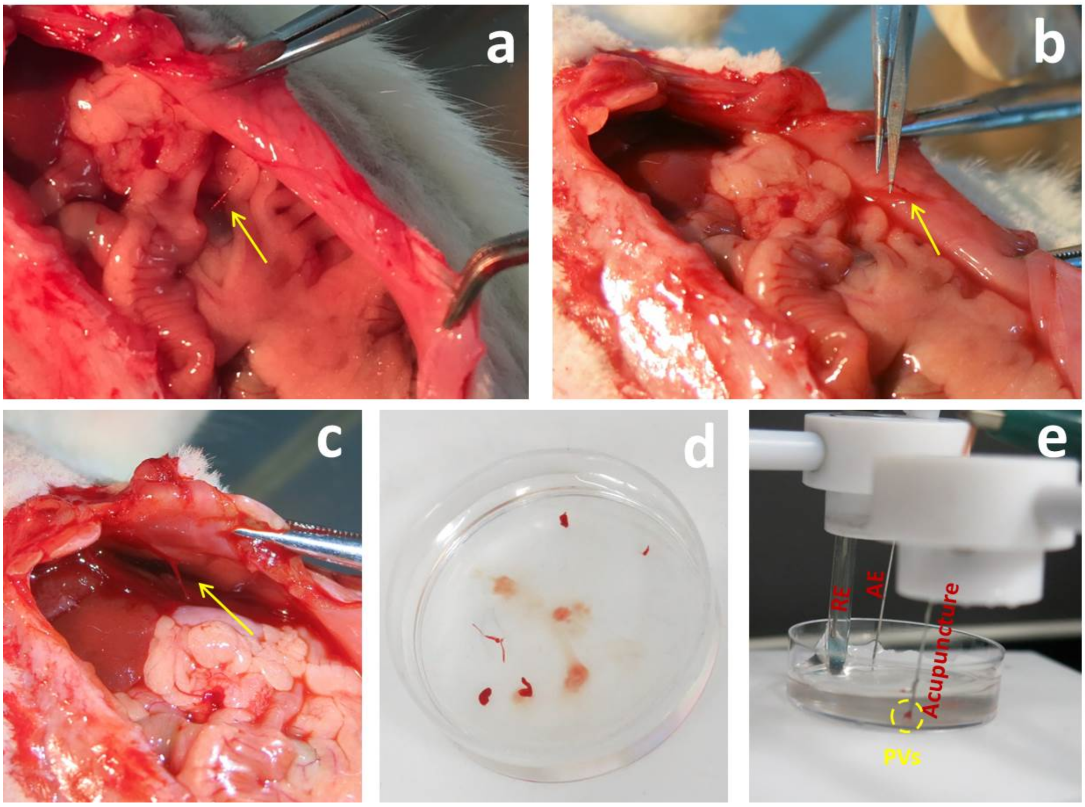

Fig. S3. (a), (b) and (c): anatomical images of typical PVs and a corpuscle (arrow), showing the PVs on the surface of intestine in SD rats. (d) Collected PVs in PBS solution. (e) Picture of real time monitoring of 5-HT in primo nodes tissue by three-electrode system.

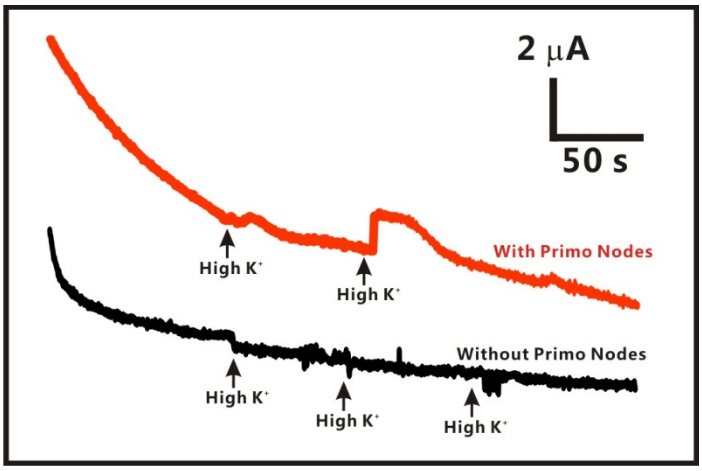

Fig. S4. Real-time monitoring of 5-HT released from primo nodes tissue in PBS solution by high K^+ stimulation.
